# Ensuring quality of health workforce education and practice: strengthening roles of accreditation and regulatory systems

**DOI:** 10.1186/s12960-020-00517-4

**Published:** 2020-10-20

**Authors:** William Burdick, Ibadat Dhillon

**Affiliations:** 1 Foundation for Advancement of International Medical Education and Research, Philadelphia, PA United States of America; 2 Health Workforce Department, World Health Organization, Geneva, Switzerland

## Abstract

Regulation of the health workforce and accreditation of educational institutions are intended to protect the public interest, but evidence of the impact of these policies is scarce and occasionally contradictory. The body of research that does exist primarily focuses on policies in the global north and on the major health professions. Stress on accreditation and regulatory systems caused by surges in demand due to the COVID-19 pandemic, privatization of education, rising patient expectations, and emergence of new health worker categories has created urgency for innovation and reform. To understand and evaluate this innovation, we look forward to receiving manuscripts which contribute to the evidence base on the implementation, management, and impact of health worker education and practice regulation, including the intersection of education accreditation and workforce regulation policy. We particularly look forward to manuscripts from underrepresented parts of the globe and underrepresented health workforce sectors that address policy effectiveness, explore different models of regulation, and present innovations that we can all learn from.

The health workforce is an important contributor to delivering health and economic prosperity [1]. Quality, accessibility, acceptability, distribution by cadre and geography, and coordination across health cadres and with other aspects of the health system are all critical elements for an effective health workforce.

Factors that affect the quality and health workforce sustainability include who is chosen to enter the field, what they are taught, how they are taught, how they are determined to be qualified to enter the field, how they maintain and update their skills, and how they are disciplined. Health workforce accreditation and regulation systems shape all of the above. Health professional regulatory processes have also been used to give effect to broader priorities related to equitable distribution, international cooperation, dual practice, and compulsory service programs. As such, the World Health Organization’s Global Strategy on Human Resources for Health: Workforce 2030 [2] emphasizes the importance of effective health personnel regulation to achieve universal health coverage, with an important role in both optimizing the existing health workforce and in aligning investments with the current and future health workforce needs.

These attributes are themselves underpinned by systems that must assure the quality of education institutions to produce the needed health workers and the appropriate level of oversight of health occupations to ensure the public interest.

Regulatory mechanisms and resources across countries of varying income classification, however, are under stress due to the increasing volume and privatization of health professional education [3], rising importance of previously unregulated occupations [4], emergence of new occupations, emergencies and humanitarian crises, new modes and cross border service delivery (e.g. use of digital technology), accelerating international mobility of health workers (OECD 2020), and escalating patient demand and expectation.

The COVID-19 pandemic has further highlighted the importance of strong and dynamic health workforce-related regulatory systems. The pandemic has stressed health workforces in waves across the world as cases and deaths surged in different locations. Alongside the supply chains and beds, health workers were in short supply or will be in short supply as surges in the number of cases continue to occur. In response, health professional regulations and associated processes were rapidly modified in many jurisdictions to expand the workforce by temporarily expanding scopes of practice and modification of professional titles, enabling the practice of retired and foreign health workers, and earlier clinical service by students [5]. In some settings, the curriculum for student health workers was modified to better prepare the workforce for the pandemic [6]. The COVID-19 pandemic brought into relief both the importance of and existing gaps in health workforce regulatory systems. Lessons learned from these efforts are important to share.

While jurisdictions attempt to assure education quality through accreditation, the evidence is sparse, difficult to generate, and sometimes contradictory. Accreditation systems may hold institutions accountable for various elements of the education system but may also inhibit innovation and can divert resources from other worthy efforts to improve the availability and quality of health services. Similarly, regulatory systems intended to protect the public can unduly inhibit prospective individuals from entering the workforce and may impose a burden that may not advance wellness or quality [7, 8].

Several factors in health workforce accreditation and regulation affect access. One is the local delineation of the “public interest” which these constructs are intended to serve. The interplay between market forces, political interests, and health workforce regulation determines many of these attributes. Entry into the field is often controlled by a licensing process that involves documentation of education and individual assessment of knowledge and skill, combined with evidence of good standing in the community. Retention of the license in many jurisdictions is contingent on evidence of continued education and re-examination of knowledge or skills. In some fields, passing a certifying examination is sufficient. Finally, the public sector or publicly sanctioned entities pass judgment based on standards for the education systems in a process known as accreditation. We would like to encourage development and publication of the evidence examining the impact of these regulations on the expressed “public interest.”

Moreover, the goals for education accreditation and workforce regulation overlap, or should overlap—regulatory thresholds for knowledge and skill should be achieved by students during the education process. That education process is evaluated by a system that is hopefully aligned with that of licensing, ensuring a smooth transition from student to practitioner.

We hope to address many of these issues in this thematic series. Our aims are to:

1. Identify empirical evidence on the impact of accreditation of education institutions on improving the quality of health worker education
2. Identify empirical evidence on the impact of health professional regulation on patient safety, quality, and broader health system objectives
3. Identify innovations in the professional regulation of health workers and the underlying drivers for reform
4. Explore the link between accreditation of education institutions and the broader regulation of professional practice
5. Provide an opportunity to present low- and middle-income country processes and practices in accreditation and health professional regulation that are currently underrepresented in the literature
6. Fill the gap in regulatory and accreditation data, evaluation, and research about health occupations such as accelerated medically trained clinicians, community health workers, dental assistants, optometric technicians, and other health occupations under-represented in the literature

In this thematic series, we are particularly interested to receive manuscripts which contribute to the evidence base on the implementation, management, and impact of health worker education and practice regulation. Manuscripts should be nationally or internationally policy-relevant. Submissions should address one or more of the following:

- Diversity of national, multi-national, and sub-national approaches to accreditation and regulation
- Facilitators and barriers to effective regulation and accreditation
- Societal impact of accreditation and regulation
- Implementation challenges for accreditation and regulation-related laws and policies
- Data sharing on implementation and impact of regulation and accreditation

Few publications address the overlapping goals of education accreditation and practice regulation [8], and we hope this series motivates researchers, analysts, professional bodies, and policymakers to generate this evidence. Diversity of approaches to the challenges of accreditation and regulation will be emphasized. The series has the potential to provide much-needed evidence for the efficacy of health workforce education and practice policies.

Finally, accreditation and regulatory research priorities were developed by a consensus process in a paper by Ranson et al. in 2010 [9]. While the priorities may need to be updated with a similar process, the list appears relevant. The priorities included the following: how effective are accreditation interventions in improving performance, how effective is re-licensing in improving health worker performance, what are the relative strengths and weaknesses of different models for regulating the private sector in LMICs, how can regulatory bodies be made more effective in regulating practice, and what is the optimal mix of financial, regulatory, and non-financial policies for improving the distribution of health workers? We hope that researchers will consider these questions for inclusion in this series.

## Data Availability

Not applicable
